# The Patriarch’s Letter

**Published:** 1857-08

**Authors:** Grant Thorburn

**Affiliations:** New Haven, Conn.


### THE PATRIARCH'S LETTER.

Doctor Hall:—I received your June Journal yesterday Thanks for your kind attention. How old are you? where did your father live sixty years ago? Where were you born? With my soul, body and spirit, I subscribe to every letter in your first article in this number, *Disease and Benevolence.* I was intimate with the Misses Jay and Gelston, fifty-five years ago. John Jay and family prayed to God in old Trinity. David Gelston and family, Robert Lenox, Senior, and family, prayed to God in the old Wall Street Church, Dr. Rodgers, Pastor; while Dr. John M. Mason prayed, and I read, line by line, and set the tune to the old Scottish version of David's Pslams, as sung by the Covenanters, in sixteen hundred and sixty-six. Dr. Mason's church was in Cedar Street, between Nassau and Broadway.

I landed in New York in June, in seventeen hundred and ninety-four; Dutch laws, Dutch language, Dutch customs, and Dutch preaching reigned paramount. I was much annoyed by the tooth-ache. The Dutch vrows advised me to smoke the pipe. It eased the pain. So I have continued to smoke five pipes every twenty-four hours, to the present day. Dr. Branbread says that tobacco is a slow poison; in my case he is correct.

I never was drunk in my life. Having cleared my dinner-plate of one-half its contents, I walk in the yard, smoke ten minutes, and cut wood with a buck and saw, fifteen. In long days and warm weather, sleep from two to three, P. M. Retire at nine, and rise at six. I have been only ten days confined to bed by indisposition, during the sixty-four years just passed.

I drink one pint of coffee with sugar, no milk, at breakfast, one pint without milk or sugar at dinner, and one pint through the afternoon and evening. I never drink cold water, and seldom swallow anything hotter than blood. I walk without a staff. I sleep without rocking, and eat my food without brandy, or bitters. I know what kills one man, may cure another, but such has been my manner of life during twenty years, just past.

All the hospitals and institutions for mitigating the miseries of man, are the fruits of Christianity. Hume, Gibbon, Voltaire, and Fanny Wright, never gave a dollar to any useful purpose, as far as I know.

N. B.—When you see my young friends, Doctors Francis and Mott, tell them I am well, and prefer beefsteak to Brandreth's pills.

I am now living with my third wife, a buxom Yankee lass, of forty-two summers, thus meeting me half way. She is a daughter of the Puritans, a lady by birth, education, and refinement. She reads Shakespeare, as I think, better than Fanny Kemble. This day, twelfth June, we are four years married, with the honeymoon still in the ascendant.

GRANT THORBURN.

*New Haven, Conn., June,* 1857.

Thus writes the hero of "*Laurie Todd,*" in his eighty-fifth year, in a bold, clear hand, every letter fully formed, every word correctly spelled, and every punctuation well placed. With a heart so joyous, on good foundation, and a spirit so buoyant, may we enter the frosty years, a full half-century hence.

